# Delusional parasitosis disorder or the Ekbom syndrome, in relation to a case

**DOI:** 10.1192/j.eurpsy.2025.2259

**Published:** 2025-08-26

**Authors:** L. Del Canto Martinez, C. Rodríguez Valbuena, G. Lorenzo Chapatte, M. Ríos Vaquero, L. Rojas Vázquez, A. Monllor Lazarraga, L. Sobrino Conde, M. Fernández Lozano, N. Navarro Barriga, B. Rodriguez Rodriguez, F. J. González Zapatero, M. J. Mateos Sexmero, A. Aparicio Parras, M. P. Pando Fernández, P. Martínez Gimeno, M. A. Andreo Vidal, M. Calvo Valcárcel, M. D. L. Á. Guillén Soto, M. E. Espinosa Muth

**Affiliations:** 1 Hospital Clínico Universitario de Valladolid, Valladolid, Spain

## Abstract

**Introduction:**

Ekbom syndrome, also known as delusional parasitosis, is a psychiatric disorder in which the affected person is firmly convinced that their body is infested with parasites, insects, or any other microorganism, despite the lack of medical evidence to support it.

A 56-year-old woman presents to the emergency department, referred by her primary care physician, due to a sensation of worms in her vagina and rectum. She reports that larvae are coming out of her nostrils, ears… and she feels them settling in her kidney. She is accompanied by her husband, who mentions that on some occasions, she has shown him the supposed parasite.

**Objectives:**

The objectives of this clinical case are to understand whether Ekbom syndrome can be related to any secondary organic pathology, as well as to identify the conditions with which the differential diagnosis should be made, and to determine the most effective treatments.

**Methods:**

Examination: Sensory-perceptual disturbances in the form of cenesthetic hallucinations. High levels of anxiety with functional impact on her daily life.

Complementary tests: A referral was made to Internal Medicine to rule out the presence of parasites, and to Neurology for an MRI with contrast, which revealed a white matter lesion in the brainstem. Tests for anti-AQP4 and anti-MOG antibodies were also conducted, and both were negative. After these studies, it was concluded that the criteria for secondary Ekbom syndrome due to organic pathology were not met.

**Results:**

The differential diagnosis should be made with other psychiatric disorders such as schizophrenia, major depression, or substance-induced psychosis. Neurological diseases, such as dementia, multiple sclerosis, or meningoencephalitis, should also be ruled out. Lastly, it is advisable to rule out dermatological conditions, such as scabies.

Treatment was initiated with Risperidone and Alprazolam, with good response and improvement in delusional ideation.

**Conclusions:**

Treatment with atypical antipsychotics is effective for this syndrome, reducing delusional ideas and significantly improving the psychiatric symptoms that accompany the delusion, such as anxiety, depression, and insomnia.

Good collaboration between different medical professionals is essential to rule out associated secondary pathology.

**Disclosure of Interest:**

None Declared

